# Association between *ADAM33* polymorphisms and asthma risk: a systematic review and meta-analysis

**DOI:** 10.1186/s12931-019-1006-1

**Published:** 2019-02-21

**Authors:** Hui-fang Li, Li-ping Yan, Kun Wang, Xiao-tong Li, Hai-xian Liu, Wei Tan

**Affiliations:** 1 Postgraduate Department of Internal Medicine, Weifang Medical University, Weifang, 261053 China; 2 Department of Human Resource Department, Weifang People’s Hospital, Weifang, 261041 China; 3 Department of Respiratory Medicine, Weifang People’s Hospital, Weifang, 261041 Shandong China

**Keywords:** Asthma, *ADAM33*, Polymorphism, Meta-analysis

## Abstract

**Background:**

Asthma is a common complex chronic, inflammatory polygenic disease with heterogeneous manifestations, affecting individuals of all age groups and posing an immense burden on healthcare resources. A number of studies have identified the association between a disintegrin and metalloprotease 33 (*ADAM33*) polymorphisms and asthma risk, however, the results still remain inconclusive. The objective of the present study was to identify the effect of *ADAM33* variants in asthma susceptibility.

**Methods:**

Eligible case-control studies published between January 2000 and June 2018 was searched and retrieved from online electronic databases. The odds ratio (OR) with its 95% confidence interval (CI) was employed to calculate the effect.

**Results:**

A total of 63 case-control studies were finally screened out, including 13,280 asthma patients and 13,340 controls. Eleven SNPs of *ADAM33* gene were identified. Our results detected a significant association between *ADAM33* T2, Q1, F + 1 and AA genotype of T + 1 polymorphisms and asthma risk in total population. Subgroup analysis by ethnicities showed that the alleles and genotypes of T2, Q1 and F + 1 polymorphisms were associated with asthma susceptibility among Asian populations, while V4 polymorphism was associated with asthma among Caucasian populations. Subgroup analysis by ages showed that T2, F + 1 and ST + 4 polymorphisms were associated with childhood asthma, while Q1 and V4 polymorphisms were associated with asthma risk in adults. Subgroup analysis by asthma severity showed that only the G allele of *ADAM33* T1 polymorphism was associated with the severity of asthma when compared with the controls. In addition, T2, Q1 and F + 1 polymorphisms of *ADAM33* were significantly associated with increased the asthma risk in Chinese asthma patients.

**Conclusions:**

Our results found that T2, Q1 and F + 1 polymorphisms of *ADAM33* gene might contribute to asthma risk. Future well-designed case-control studies with large population and more ethnicities are still needed to estimate the association.

## Background

Asthma is a lifelong respiratory disease of the lung airways, characterized by variable and recurring symptoms, reversible airflow obstruction and bronchospasm [1]. It involves inflammation and swelling of the airways, resulting in permanent remodeling/scarring of the airways. The symptoms of asthma include episodes of wheezing, coughing, chest tightness, and shortness of breath, which may occur a few times a day or a few times per week [2]. Depending on the individuals, the symptoms may become worse at night or with exercise, posing significant burden on quality of life, work productivity, activity impairment, and healthcare resources use [3]. Both the genetic factors (genetic polymorphisms, family history) and environmental factors (tobacco smoke, air pollution, allergens) contribute to the development of asthma [4, 5]. Prevalence rates increase and mortality rates decline for asthma in many countries over the past few decades: in 2016, 360 million people globally had asthma, up from 183 million in 1990 [6]; an estimated 420,000 people died from asthma worldwide in 2016, compared to 474,000 in 1990. Although many medicines are now widely used due to their greater effectiveness and fewer side effects in treatment of asthma, unfortunately, none of these medications are available without a prescription [7, 8]. In addition, a remarkably high frequency of poorly controlled asthma has been identified, and for some 5–10% of asthma patients, the disease is refractory to corticosteroid treatment and often leads to hospital admissions caused by respiratory viral infection with rhinovirus. What’s more, asthma symptoms beginning in adults might have originated in childhood [9], and if lung function no longer returns normal when not having an attack, asthma moves into the category of disease known as chronic obstructive pulmonary disease (COPD). Therefore, there is an urgent need to identify some significant biomarkers which can predict this disease and guild the therapeutic strategies.

A large number of genetic studies have been conducted to identify genetic polymorphisms associated with asthma susceptibility. A disintegrin and metalloprotease 33 (*ADAM33*) gene, located on human chromosome 20p13, was one of the first asthma candidate genes identified by positional cloning. It is one member of the ADAM family of zinc-dependent metalloproteases, and plays an important biological role as an activator of growth factors and Th2 cytokines [10]. *ADAM33* consists of 22 exons that encode a signal sequence, pre-domain, catalytic domain, disintegrin domain, cysteine-rich domain, EGF domain, transmembrane domain, and cytoplasmic domain with a long 3′-untranslated region (UTR) [11]. These different domains translate into different biological functions of *ADAM33*, involving cell activation, proteolysis, adhesion, fusion, and intracellular signaling [12]. Genetic studies have demonstrated that *ADAM33* may be involved in determining lung function throughout life, associated with increased the risk of therapeutic intervention decline in asthma [13]. Soluble ADAM33 (sADAM33) is identified to promote angiogenesis, defining it as a tissue remodeling gene with potential to affect airflow obstruction and lung functions independently of inflammation [14]. Evidences have shown that *ADAM33* functions as a susceptibility target gene for asthma [15], and has an important role in the natural history and possibly the origins of asthma [16]. Besides, the preferential expression of *ADAM33* mRNA in smooth muscle, fibroblasts, and myofibroblasts suggest that the abnormalities of its function may link to bronchial hyperresponsiveness (BHR) and airway wall “remodelling” which contributes to the early life origins of asthma. Moreover, a higher expression of ADAM33 protein was detected in asthma patients compared to controls [17]. The ADAM33 expression was also increased as asthma severity increases, which may contribute to the remodeling process that occurs with asthma progression and may have implications for future treatment in severe disease [18]. Genetic polymorphisms might alter the gene function, thus playing a role in disease progression. At present, more than 100 single-nucleotide polymorphisms (SNPs) of the *ADAM33* gene have been reported to be associated with asthma and related traits: V4 (rs2787094, 3’UTR, C/G), T + 1 (rs2280089, intron, G/A), T2 (rs2280090, cytoplasmatic domain, G/A), T1 (rs2280091, cytoplasmatic domain, A/G), S2 (rs528557, transmembrane domain, G/C), S1 (rs3918396, transmembrane domain, G/A), Q1 (rs612709, intron, G/A), F + 1 (rs511898, intron, C/T), S + 1 (rs2853209, intron, A/T) and so on. Several polymorphic sites were shown to be associated with asthma risk in different populations [19].

A serial of case-control studies have identified the role of *ADAM33* polymorphisms in asthma risk, however, the results still remain inconclusive. For example, Awasthi et al. demonstrated that the V4 C/G of the *ADAM33* gene was associated with asthma risk [20], while Anand et al. found that there was no association of *ADAM33* V4 polymorphism with asthma risk [21]. Fedorova et al. detected a significant association between A allele of ST + 4 and asthma susceptibility in Russian patients [22], while Dmitrievazdorova et al. found that probably *ADAM33* ST + 4 gene was not directly involved in predisposition to asthma in Russian patients [23]. In addition, asthma is a heterogeneous disorder with a complex etiology, and the frequency and severity of asthma differs between different racial and ethnic groups, ages, and economic conditions [24, 25]: the average annual asthma prevalence is higher in children (9.5%) than adults (7.7%); the prevalence of asthma is higher in black persons than white persons; asthma prevalence increases with each successive lower poverty level group [26]. Therefore, we conducted the present meta-analysis to evaluate the *ADAM33* gene polymorphisms in asthma risk based on all the available published data to obtain a more reliable result.

## Methods

### Literature search and identification of eligible studies

A comprehensive literature search was conducted using the electronic databases of English (PubMed, Embase, LISTA, Web of science and Library of Congress) and Chinese (Wanfang and China National Knowledge Infrastructure) to search relevant articles published between January 2000 and June 2018. The Medical Subject Heading (MeSH) terms were: “asthma or asthmatic”, “a disintegrin and metalloprotease 33 or *ADAM33*”, and “polymorphism or SNP or variant”. The equivalent Chinese words were used in the Chinese databases. The references of retrieved articles were manually checked to obtain more data. When the same department published more than one article on the same subject among the same participants, only the most recent full-text article was included into the final analysis.

### Inclusion and exclusion criteria for article screening

Eligible articles must meet the following inclusion criteria: 1) case-control study evaluating the association between *ADAM33* polymorphisms and asthma susceptibility; 2) the patients were diagnosed with asthma by chest physicians according to Global Initiative for Asthma (GINA) guidelines [27]; 3) the controls were age-matched unrelated participants of the same ethnicity with no history of asthma or other chronic pulmonary diseases; 4) the alleles and genotypes for each SNP were available to extract, and the results were expressed as odds ratio (OR) with its corresponding 95% confidence interval (CI); and 5) the genotypes in controls must be consistence with the Hardy-Weinberg equilibrium (HWE, *P* > 0.05). The exclusion criteria were: 1) conference papers, reports, comments or review articles; 2) with duplicated data; 3) without the control group; and 4) the data couldn’t be extracted.

### Quality assessment and data extraction

Two of our authors independently estimated the information extracted from case-control studies based on our inclusion and exclusion criteria. Any disagreement was resolved by discussing with the third author to reach a final consensus. The following information was retrieved: the name of first author, publication year, country, ethnicity, mean age, number of cases and controls, genotyping methods and SNPs.

### Statistical analysis

All the statistical analyses were carried out using the RevMan software (version 5.3). The OR with 95% CI was employed to calculate the association between *ADAM33* polymorphisms and asthma risk. The pooled OR was determined using the Z test with *P*-value less than 0.05 (considered as significant). For each SNP, the allelic model, homozygous model, heterozygous model, dominant model and recessive model were examined to evaluate its effect. For subgroup analysis, only the allele and genotype differences from control subjects were analyzed. The between-study heterogeneity was assessed by the Q test and the I^2^ test. The fixed-effect model was used when the *P*-value of the Q test was more than 0.10 and I^2^ of the I^2^ test was less than 50%; otherwise, the random-effect model was used. To estimate whether our results were substantially influenced by the presence of any individual study, a sensitivity analysis was performed by systematically omitting each included case-control study and recalculating the significance of the result. The funnel plot was used to examine the publication bias. In the absence of publication bias, it assumes that studies with high precision (large study effects) will be plotted near the average, and studies with low precision (small study effects) will be spread evenly on both sides of the average, creating a roughly funnel-shaped distribution. Deviation from this shape can indicate publication bias.

## Results

### Main characteristics of included studies

**Fig. 1:**
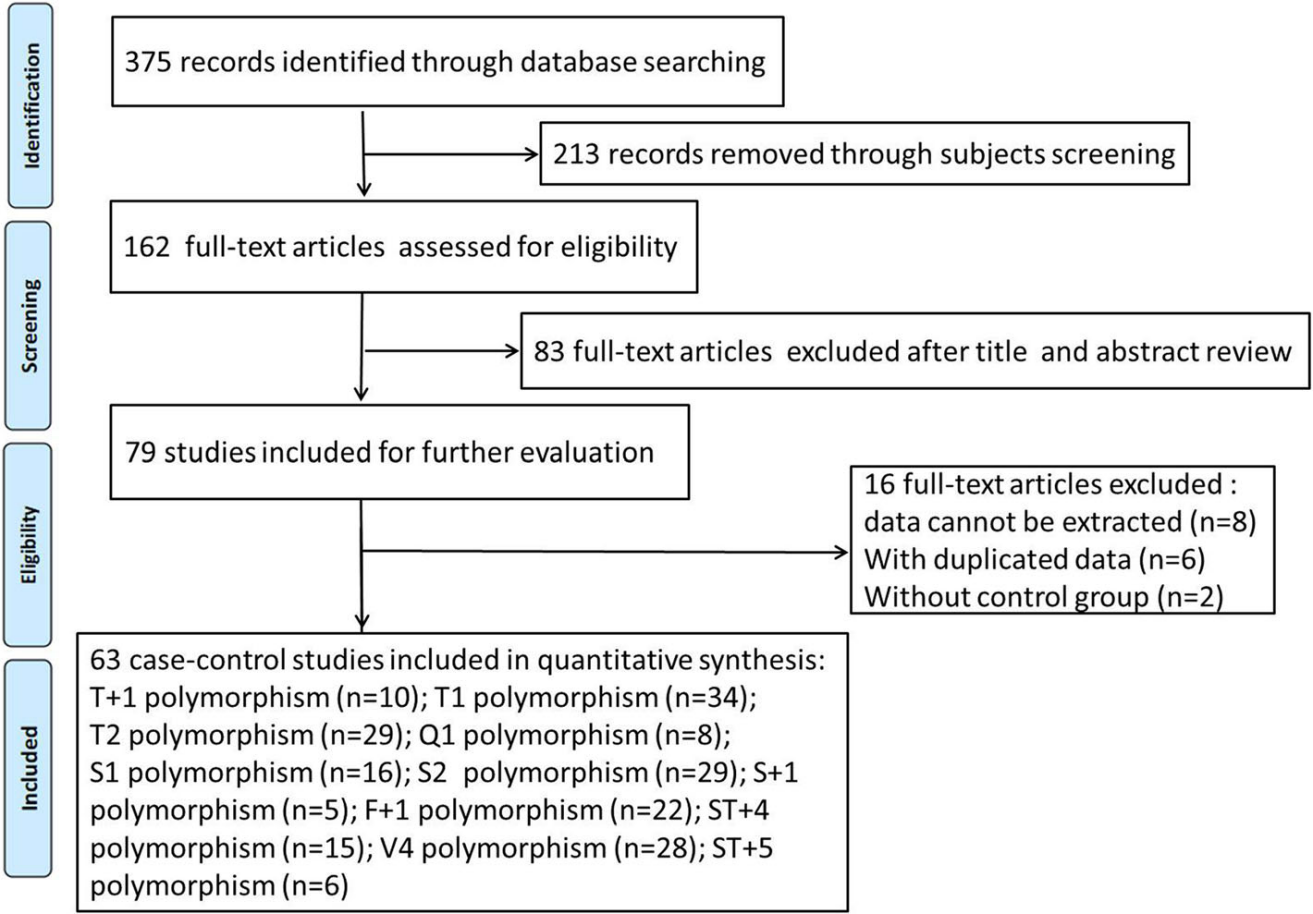
Flow chart of literature search and study selection strategy for included articles in the meta-analysis

According to combination of the key searching MeSH terms and the online databases, we firstly obtained 375 available case-control studies. After applying the inclusive and exclusive criteria, a total of 63 articles were finally screened out, including 13,280 asthma patients and 13,340 controls. Figure 1 showed the flow chart of literature search and study selection. The 63 included articles were conducted in twenty countries: China [28–61], Korea [62], Japan [63, 64], Germany [65], Thailand [66], Brazil [67], India [68–73], Colombia [74], Saudi Arabia [75], Czech Republic [76], Turkey [77], Egypt [78, 79], Portugal [80], Jordan [81], Iran [82–84], Pakistan [85–87], Iraq [88], Venezuela [89], Mixed (Dutch and America) [90]. Of the retrieved articles, forty-one were written in English and twenty-two were in Chinese; fifty-three were performed in Asian populations, seven were in Caucasian populations and two were in African populations; twenty-one were conducted in children, fifteen were in adults, and others were in mixed populations or not mention the study populations. The sample size ranged from 89 to 1872. 11 SNPs of *ADAM33* gene were finally identified: T + 1 (rs2280089, G/A), T1 (rs2280091, A/G), T2 (rs2280090, G/A), Q1 (rs612709, G/A), S1 (rs3918396, G/A), S2 (rs528557, G/C), S + 1 (r2853209, A/T), F + 1 (rs511898, C/T), ST + 4 (rs44707, C/A), ST + 5 (rs597980, C/T) and V4 (rs2787094, C/G). The main characteristics of included studies were presented in Table 1.

**Table 1:** Main characteristics of included case-control studies in this meta-analysis

First author	Year	Country	Ethnicity	Study population	Mean age		Sample size		Genotyping Methods	SNPs
					Cases	Controls	Cases Controls			
Howard TD	2003	Mixed	Mixed	NA	NA	NA	667	752	MassARRAY	S1,S2,T1,T2,V4,ST + 4
Lee JH	2004	Korea	Asian	Mixed	48(11–78)	27(10–74)	326	151	SNaPshot	S1,T1,V4
Hirota T	2006	Japan	Asian	Adults	48.7 (18–91)	44 (18–83)	504	651	Taqman/PCR-RFLP	F + 1,T1,T2,ST + 4,V4,S2
Schedel M	2006	Germany	Caucasian	Children	9–11	9–11	624	1248	MALDI-TOF	F + 1,S1,S2,ST + 4,T1,T2,V4
Qiu YM	2007	China	Asian	Mixed	39.88 ± 13.29	43.00 ± 12.55	160	95	PCR-RFLP	T1
Wang P	2006	China	Asian		43.43	41.91	296	270	PCR-RFLP	F + 1,S + 1,T1
de Faria ICJ	2008	Brazil	Caucasian	Children	10.3 ± 2.79	34 ± 11.3	88	202	PCR-RFLP	S2
Hu ZL	2008	China	Asian	Mixed	42.88 (13–75)	42.95 (18–76)	147	73	Sequencing	S1,S2
Su DJ	2008	China	Asian	NA	36.69 ± 11.53	37.18 ± 10.60	181	151	PCR-RFLP	V4,T + 1,T1,T2,S1,Q1
Thongngarm T	2008	Thailand	Asian	NA	29.86	26	200	100	Sequencing	S2,V4,ST + 4
Xiong JY	2009	China	Asian	Children	6–13	6–13	92	91	PCR-RFLP	T1
Ye H	2009	China	Asian	Adults	44.42(18–70)	43.26 (22–64)	186	150	Sequencing	T2,S2
Bijanzadeh M	2010	India	Asian	Mixed	0.5–80	0.5–80	100	50	PCR-RFLP	T1
Vergara CI	2010	Colombia	Caucasian	Adults	36.15 ± 18.32	34.98 ± 17.8	429	401	Taqman	S2,T1,T2,V4
Awasthi S	2011	India	Asian	Children	1–15	1–15	211	137	PCR-RFLP	F + 1,S2,V4,ST + 4
Jie ZJ	2011	China	Asian	Adults	37.1 (20–69)	43.7 (20–69)	150	74	PCR-RFLP	F + 1, S1,S2,T1,T2,ST + 4
Jin JW	2011	China	Asian	Adults	34.3 ± 12.94	35.85 ± 2.26	116	70	Sequencing	T1
Qu SQ	2011	China	Asian	Children	7.74 ± 2.78	7.52 ± 2.95	412	397	PCR-RFLP	F + 1,T + 1,T1,T2,V4,Q1
Ren L	2011	China	Asian	Children	8.22 ± 2.37	8.17 ± 3.09	308	251	MALDI-TOF	F + 1,Q1,S2,T1,T2,V4,ST + 4
Tripathi P	2011	India	Asian	Adults	33.7 ± 11.3	31.9 ± 9.2	175	253	PCR-RFLP	F + 1,V4,S2,ST + 4
Yang ZH	2011	China	Asian	NA	NA	NA	100	144	PCR-RFLP	S2,T2
Yu HC	2011	China	Asian	Children	3.9 ± 3.1	3.3 ± 2.7	122	137	PCR-RFLP	T1
Al-Khayyat AI	2012	Saudi Arabia	Asian	Children	3–12	3–12	107	87	Taqman, PCR-RFLP, ARMS-PCR	T1,T2,ST + 4,S1
Bora E	2012	Turkey	Caucasian	Children	9.30 ± 2.93	10.8 ± 3.14	98	100	BioEdit	T1,T2,T + 1
Chi XY	2012	China	Asian	Mixed	39.88 ± 13.29	43.00 ± 12.55	126	121	AS-PCR	F + 1,T2,V4,S2
Chiang CH	2012	Taiwan	Asian	NA	46 ± 20	44 ± 17	476	115	Taqman	T1,
Godava M	2012	Czech Republic	Caucasian	Children	0.4–18	0.4–18	109	45	Taqman	F + 1,S1,S2,S + 1,ST + 4,T1, T + 1,V4
Miyake Y	2012	Japan	Asian	Adults	30.4 ± 4.2	31.5 ± 4.2	89	1281	Taqman	V4,T + 1,T2,T1,S + 1,S2,Q1
Tripathi P	2012	India	Asian	Mixed	18.7 ± 15.9	22.9 ± 14.5	386	390	PCR-RFLP	T + 1,S + 1
Wei X	2012	China	Asian	Mixed	32.73 ± 9.29	30.55 ± 5.32	70	89	PCR-RFLP	T1,V4
Zhao B	2012	China	Asian	Children	14.2 ± 3.4	14.5 ± 3.5	110	144	PCR-RFLP	V4,T2
El-Falaki MM	2013	Egypt	African	Children	3–12	3–12	60	32	PCR-RFLP	T1,T2,V4
Xie CL	2013	China	Asian	NA	64.1 ± 10.2	64.3 ± 10.5	220	300	Taqman	S1,S2,T1,T2
Xue WL	2013	China	Asian	Adults	49.7 (18–65)	39.5 (18–65)	400	200	Sequencing	T2,F + 1,V4,S2,ST + 4
Berenguer AG	2014	Portugal	Caucasian	Children	13.6 ± 4.3	NA	98	105	Taqman	V4,S1
Li HB	2014	China	Asian	Children	10.6 ± 2.7	10.4 ± 2.9	299	311	PCR-RFLP	V4,T2,S2,T1
Karimi MRZ	2014	Iran	Asian	Mixed	37 (7–64)	39 (2–75)	95	86	PCR-RFLP	S1,T1,T2,V4
Wang J	2014	China	Asian	Mixed	41.06 ± 9.75	40.58 ± 9.24	126	126	PCR-RFLP	T1,T2,V4
Yao LD	2014	China	Asian	Mixed	41.06 ± 9.75	40.58 ± 9.24	126	126	PCR-RFLP	F + 1,S + 1
Yilihamu N	2014	China	Asian	Adults	42.93 ± 13.48	41.14 ± 14.07	183	155	PCR-RFLP	T2
Zihlif M-1	2014	Jordan	Asian	Children	5.96 ± 4.63	7.53 ± 4.83	107	115	PCR-RFLP	T1,T2,T + 1,V4,S1,S2,F + 1,Q1
Zihlif M-2				Adults	44.7 ± 15.41	38.4 ± 14.85	160	110		
Fan JG	2015	China	Asian	Children	5.27 ± 1.93	5.68 ± 2.17	120	105	PCR-RFLP	F + 1,T1,S2
Gong L	2015	China	Asian	NA	34.5 ± 13.2	36.5 ± 11.4	100	100	PCR-RFLP	F + 1,S2
Qiao LP	2015	China	Asian	Adults	18–74	20–69	295	255	AS-PCR/Sequencing	T2
Khadhim MM	2015	Iraq	Asian	Adults	18–70	18–69	69	20	PCR-RFLP	V4
Raja GK	2015	Pakistan	Asian		NA	NA	298	204	PCR-RFLP	V4,S1
Sabar MF	2015	Pakistan	Asian	Mixed	21.59 ± 15	30.15 ± 9.16	101	102	SNaPshot	T + 1,T2,S2,Q1,F + 1,ST + 4,T1
Sinha S	2015	India	Asian	NA	37.22 ± 14.1	34.29 ± 12.2	481	483	PCR-RFLP	F + 1,V4
Shalaby SM	2016	Egypt	African	Children	8.5 ± 3.6	8.8 ± 2.6	400	200	PCR-RFLP	F + 1,ST + 4
Awasthi S	2016	India	Asian	NA	18.7 ± 15.9	22.87 ± 14.54	386	390	PCR-RFLP	T2,T1,Q1,S1
Hu X	2016	China	Asian	NA	42.89 ± 14.03	40.22 ± 14.56	206	140	PCR-RFLP	S2,ST + 4
Liang SQ	2016	China	Asian	NA	37.69 ± 12.23	34.67 ± 11.46	107	119	PCR-RFLP	S2,T2
Martínez D	2016	Venezuela	Caucasian	NA	41 ± 16	34 ± 12	103	100	PCR-RFLP	V4,T1
Wang JR	2016	China	Asian	Children	8.15(5.98–10.16)	7.68 (5.67–10.04)	197	120	PCR-RFLP	V4,T2
Zhang LH	2016	China	Asian	Children	7.89 ± 3.08	8.16 ± 3.11	153	103	MassARRAY	T2,V4,F + 1,S2,ST + 4
Huang Y	2017	China	Asian	Adults	41.69 ± 10.87	41.06 ± 10.41	39	50	SNaPshot	F + 1,S2
Lv JS	2017	China	Asian	Children	5–15	5–15	154	120	PCR-RFLP	V4
Shen B	2017	China	Asian	Mixed	22.37 ± 19.72	21.71 ± 20.64	150	100	PCR-RFLP	T2,F + 1,S2,T + 1,T1,V4,S1,Q1
Yu SL	2017	China	Asian	Children	7.19 ± 2.93	7.73 ± 2.57	120	120	PCR-RFLP	T2
Zeinaly I	2017	Iran	Asian	NA	25.84 ± 15.08	27.43 ± 15.64	190	180	PCR-RFLP	T1,V4
Zhu SF	2017	China	Asian	Adults	44 ± 12.7	42 ± 10.9	215	220	PCR-RFLP	T1,T2,V4,S2
Farjadian S	2018	Iran	Asian	Mixed	5–82	5–82	150	149	PCR-RFLP	T + 1,T1,S1,F + 1
Saba N	2018	Pakistan	Asian	NA	40 ± 0.93	30 ± 0.97	333	200	iPLEX	T1,S2

*NA* not applicable, *SNPs* single nucleotide polymorphisms, *PCR-RFLP* polymerase chain reaction-restriction fragment length polymorphism, *AS-PCR* Allele-specific PCR, *MALDI-TOF* matrix-assisted laser desorption/ionizationtime-of-flight mass spectrometry

### Meta-analysis of association between *ADAM33* genetic polymorphisms and asthma risk

If the number of available case-control articles for each SNP or subgroup analysis was equal to or more than three, we will conduct the statistical analyses. Table 2 listed the meta-analysis results of association between *ADAM33* genetic variants and asthma risk in total population and subgroup analysis by ethnicities and ages.

**Table 2:** Meta-analysis of the association between ADAM33 genetic polymorphisms and asthma risk in total population and subgroup analysis by ethnicities and ages

SNPs	Comparisons	N	Test of association		Test of heterogeneity		
			OR (95%CI)	P	Ph	I^2^(%)	Model
T + 1 (rs2280089)							
G/A	A vs. G	10	1.18 (0.87, 1.60)	0.27	< 0.00001	80	R
	AA vs. GG		1.55 (1.01, 2.38)	0.04	0.44	0	F
	AG vs. GG		1.23 (0.87, 1.74)	0.24	< 0.00001	79	R
	AA+AG vs. GG		1.23 (0.87, 1.74)	0.25	< 0.00001	80	R
	AA vs. AG + GG		1.37 (0.89, 2.10)	0.15	0.55	0	F
Asians	A vs. G	8	1.32 (0.96, 1.81)	0.09	< 0.0001	80	R
	AA+AG vs. GG		1.38 (0.96, 1.99)	0.08	< 0.00001	80	R
Children	A vs. G	4	1.16 (0.54, 2.49)	0.71	< 0.00001	90	R
	AA+AG vs. GG		1.17 (0.51, 2.69)	0.71	< 0.00001	89	R
T1 (rs2280091)							
A/G	G vs. A	34	1.10 (0.90, 1.34)	0.34	< 0.00001	88	R
	GG vs. AA		1.24 (0.83, 1.86)	0.30	< 0.00001	71	R
	AG vs. AA		1.13 (0.91, 1.39)	0.26	< 0.00001	82	R
	GG + AG vs. AA		1.14 (0.91, 1.43)	0.26	< 0.00001	87	R
	GG vs. AG + AA		1.15 (0.83, 1.60)	0.40	< 0.00001	65	R
Asians	G vs. A	27	0.83 (0.65, 1.05)	0.13	< 0.00001	89	R
	GG + AG vs. AA		1.24 (0.94, 1.65)	0.13	< 0.00001	88	R
Caucasians	G vs. A	6	1.11 (0.97, 1.28)	0.14	0.15	38	F
	GG + AG vs. AA		0.89 (0.76, 1.05)	0.16	0.12	43	F
Children	G vs. A	12	1.18 (0.83, 1.66)	0.36	< 0.00001	88	R
	GG + AG vs. AA		1.41 (0.98, 2.04)	0.07	< 0.00001	83	R
Adults	G vs. A	6	0.76 (0.53, 1.08)	0.13	0.0007	76	R
	GG + AG vs. AA		0.82 (0.51, 1.31)	0.40	< 0.0001	84	R
T2 (2280090)							
G/A	A vs. G	29	1.32 (1.08, 1.60)	0.006	< 0.00001	86	R
	AA vs. GG		1.77 (1.09, 2.87)	0.02	< 0.00001	68	R
	AG vs. GG		1.25 (1.03, 1.52)	0.02	< 0.00001	80	R
	AA+AG vs. GG		1.30 (1.06, 1.61)	0.01	< 0.00001	84	R
	AA vs. AG + GG		1.62 (1.07, 2.46)	0.02	< 0.00001	57	R
Asians	A vs. G	24	1.44 (1.13, 1.85)	0.004	< 0.00001	88	R
	AA+AG vs. GG		1.45 (1.11, 1.88)	0.006	< 0.00001	86	R
Caucasians	A vs. G	4	0.93 (0.80, 1.09)	0.38	0.14	45	F
	AA+AG vs. GG		0.88 (0.67, 1.15)	0.34	0.09	55	R
Children	A vs. G	12	1.45 (1.11,1.89)	0.006	< 0.00001	79	
	AA+AG vs. GG		1.43 (1.07, 1.92)	0.02	< 0.00001	78	R
Adults	A vs. G	10	1.04 (0.83, 1.29)	0.75	0.05	62	R
	AA+AG vs. GG		1.03 (0.81, 1.32)	0.79	0.004	62	R
Q1 (rs612709)							
G/A	A vs. G	8	1.26 (1.10, 1.45)	0.0007	0.13	37	F
	AA vs. GG		1.78 (1.11, 2.84)	0.02	0.54	0	F
	AG vs. GG		1.26 (0.99, 1.60)	0.06	0.04	53	R
	AA+AG vs. GG		1.27 (1.09, 1.48)	0.002	0.07	47	F
	AA vs. AG + GG		1.65 (1.04, 2.61)	0.03	0.35	11	F
Children	A vs. G	3	1.17 (0.95, 1.43)	0.13	0.85	0	F
	AA+AG vs. GG		1.19 (0.95, 1.50)	0.13	1.00	0	F
S1 (rs3918396)							
G/A	A vs. G	16	0.85 (0.55, 1.31)	0.46	< 0.00001	92	R
	AA vs. GG		0.39 (0.14, 1.13)	0.08	< 00001	83	R
	AG vs. GG		0.95 (0.71, 1.27)	0.71	0.0007	62	R
	AA+AG vs. GG		0.82 (0.54, 1.23)	0.33	< 00001	83	R
	AA vs. AG + GG		0.60 (0.28, 1.30)	0.20	< 00001	90	R
Asians	A vs. G	13	0.83 (0.48, 1.43)	0.50	< 0.00001	93	R
	AA+AG vs. GG		0.76 (0.42, 1.36)	0.35	< 0.00001	84	R
Caucasians	A vs. G	3	1.05 (0.85, 1.30)	0.66	0.12	54	F
	AA+AG vs. GG		1.07 (0.85, 1.34)	0.59	0.14	50	F
Children	A vs. G	4	1.23 (0.96, 1.57)	0.10	0.26	26	F
	AA+AG vs. GG		1.27 (0.98, 1.66)	0.07	0.35	8	F
S2 (rs528557)							
G/C	C vs. G	29	1.05 (0.86 1.29)	0.63	< 0.00001	92	R
	CC vs. GG		1.04 (0.70, 1.56)	0.83	< 0.00001	88	R
	GC vs. GG		1.08 (0.84, 1.38)	0.56	< 0.00001	82	R
	CC + GC vs. GG		1.06 (0.80, 1.40)	0.70	< 0.00001	88	R
	CC vs. GC + GG		1.04 (0.82, 1.33)	0.75	< 0.00001	85	R
Asians	C vs. G	24	1.07 (0.82, 1.41)	0.61	< 0.00001	94	R
	CC + GC vs. GG		1.09 (0.75, 1.58)	0.66	< 0.00001	89	R
Caucasians	C vs. G	5	0.96 (0.86, 1.07)	0.45	0.11	47	F
	CC + GC vs. GG		0.87 (0.63, 1.21)	0.40	0.009	71	R
Children	C vs. G	9	1.07 (0.70, 1.63)	0.75	< 0.00001	94	R
	CC + GC vs. GG		1.07 (0.57, 2.01)	0.83	< 0.00001	0	R
Adults	C vs. G	9	1.04 (0.67, 1.61)	0.87	< 0.00001	96	R
	CC + GC vs. GG		1.01 (0.59, 1.73)	0.97	< 0.00001	92	R
S + 1 (r2853209)	T vs. A	5	0.91 (0.81, 1.03)	0.14	0.99	0	F
A/T	TT vs. AA		0.82 (0.64, 1.05)	0.12	0.99	0	F
	TA vs. AA		0.99 (0.81, 1.21)	0.91	0.98	0	F
	TT + TA vs. AA		0.93 (0.77, 1.13)	0.49	0.99	0	F
	TT vs. TA + AA		0.82 (0.67, 1.02)	0.07	0.99	0	F
F + 1 (rs511898)	T vs. C	22	1.17 (1.06, 1.30)	0.002	0.0001	61	R
C/T	TT vs. CC		1.43 (1.14, 1.80)	0.002	0.001	55	R
	TC vs. CC		1.11 (1.01, 1.21)	0.02	0.55	0	F
	TT + TC vs. CC		1.15 (1.06, 1.26)	0.001	0.11	28	F
	TT vs. TC + CC		1.29 (1.05, 1.59)	0.02	0.0002	60	R
Asians	T vs. C	19	1.15 (1.03, 1.28)	0.009	0.003	54	R
	TT + TC vs. CC		1.13 (1.03, 1.24)	0.01	0.12	28	F
Children	T vs. C	8	1.26 (1.05, 1.52)	0.01	0.001	71	R
	TT + TC vs. CC		1.22 (1.07, 1.39)	0.003	0.40	4	F
Adults	T vs. C	6	1.17 (0.92, 1.48)	0.20	0.0004	76	R
	TT + TC vs. CC		1.13 (0.83, 1.55)	0.43	0.003	70	R
ST + 4 (rs44707)							
C/A	A vs.C	15	0.90 (0.80, 1.02)	0.09	0.0002	65	R
	AA vs. CC		0.82 (0.64, 1.04)	0.10	0.0009	62	R
	CA vs. CC		0.90 (0.81, 1.01)	0.07	0.15	28	F
	AA+CA vs. CC		0.88 (0.75, 1.03)	0.11	0.01	50	R
	AA vs. CA + C		0.88 (0.73, 1.05)	0.15	0.004	56	R
Asians	A vs.C	10	0.92 (0.79, 1.07)	0.28	0.005	62	R
	AA+CA vs. CC		0.88 (0.72, 1.08)	0.22	0.03	53	R
Caucasians	A vs.C	4	0.99 (0.87, 1.12)	0.82	0.72	0	F
	AA+CA vs. CC		1.00 (0.82, 1.22)	0.99	0.56	0	F
Children	A vs.C	7	0.82 (0.69, 0.97)	0.02	0.04	55	R
	AA+CA vs. CC		0.81 (0.69, 0.95)	0.010	0.28	19	F
Adults	A vs.C	4	0.83 (0.66, 1.05)	0.13	0.02	70	R
	AA+CA vs. CC		0.82 (0.60, 1.12)	0.21	0.05	63	R
V4 (rs2787094)							
C/G	G vs. C	28	1.14 (0.95, 1.36)	0.15	< 0.00001	91	R
	GG vs. CC		1.25 (0.90, 1.74)	0.18	< 0.00001	85	R
	CG vs. CC		1.16 (0.97, 1.39)	0.11	< 0.00001	74	R
	GG + CG vs. CC		1.18 (0.95, 1.47)	0.14	< 0.00001	85	R
	GG vs. CG + CC		1.17 (0.92, 1.49)	0.20	< 0.00001	84	R
Asians	G vs. C	22	1.16 (0.92, 1.46)	0.22	< 0.00001	93	R
	GG + CG vs. CC		1.19(0.88, 1.61)	0.26	< 0.00001	88	R
Caucasians	G vs. C	6	1.12 (1.00, 1.26)	0.04	0.78	0	F
	GG + CG vs. CC		1.17 (1.01, 1.36)	0.04	0.94	0	F
Children	G vs. C	12	0.99 (0.73, 1.36)	0.96	< 0.00001	92	R
	GG + CG vs. CC		0.98 (0.68, 1.42)	0.92	< 0.00001	85	R
Adults	G vs. C	7	1.39 (1.04, 1.84)	0.02	< 0.00001	85	R
	GG + CG vs. CC		1.52 (1.04, 2.21)	0.03	0.0002	77	R
ST + 5 (rs597980)							
C/T	T vs. C	6	1.21 (0.84, 1.73)	0.31	< 0.00001	91	R
	TT vs. CC		1.41 (0.75, 2.67)	0.29	< 0.00001	88	R
	TT + CT vs. CC		1.21 (0.83, 1.76)	0.32	0.0001	80	R
	TT vs. CT + CC		1.30 (0.76, 2.23)	0.34	< 0.00001	89	R

*SNPs* single nucleotide polymorphisms, *N* number of included studies, *OR* odds ratio, *95% CI* 95% confidence interval, *Ph p*-value of heterogeneity, *R* random-effect model, *F* fixed-effect model

### T + 1 (rs2280089, G/A)

Ten articles included 1943 asthma patients and 2940 controls. Eight articles were conducted in Asian population and two were in Caucasian populations. Our result found that only AA genotype was associated with asthma risk when compared with the GG genotype (AA vs. GG: OR = 1.55, 95% CI = 1.01–2.38, *P* = 0.04) in the fixed-effect model. Subgroup analysis by ethnicities showed that the A allele and AA+AG genotype of *ADAM33* T + 1 polymorphism was not associated with asthma risk in Asians population (*P* > 0.05). This genetic polymorphism did not increase the risk of childhood asthma as well (A vs. G: OR = 1.16, 95% CI = 0.54–2.49, *P* = 0.71; AA+AG vs. GG: OR = 1.17, 95% CI = 0.51–2.69, P = 0.71) in the random-effect model.

### T1 (rs2280091, A/G)

Thirty-four case-control studies included 7873 asthma patients and 8537 controls. A significant between-study heterogeneity was observed, and the random-effect model was used. Overall, we did not detect a significant association between T1 polymorphism and asthma risk in total population. Subgroup analysis by ethnicities and ages did not find a significant association between this genetic polymorphism and asthma susceptibility in any comparison groups.

### T2 (rs2280090, G/A)

**Fig. 2:**
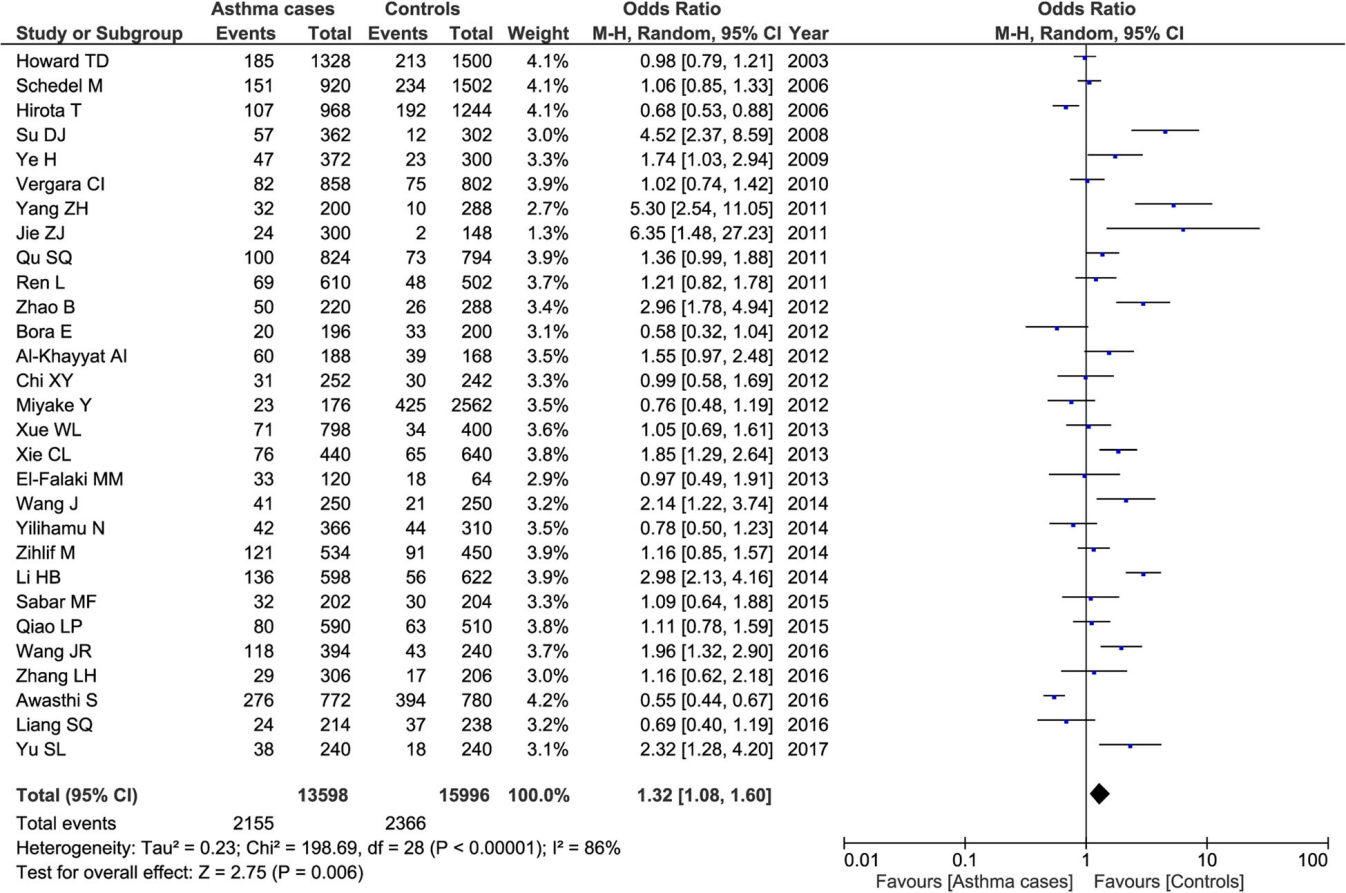
Forest plots of the association between *ADAM33* T2 polymorphism and asthma risk in total population under the allelic model

Twenty-nine case-control studies included 6799 cases and 7998 controls. Our result detected a significant association between this genetic polymorphism and asthma risk under all the genetic models (A vs. G: OR = 1.32, 95% CI = 1.08–1.60, *P* = 0.006; AA vs. GG: OR = 1.77, 95% CI = 1.09–2.87, *P* = 0.02; AG vs. GG: OR = 1.25, 95% CI = 1.03–1.52, P = 0.02; AA+AG vs. GG: OR = 1.30, 95% CI = 1.06–1.61, *P* = 0.01; AA vs. AG + GG: OR = 1.62, 95% CI = 1.07–2.46, P = 0.02). Figure 2 showed the T2 polymorphism in asthma risk under the allelic model. Subgroup analysis by ethnicities showed that both the A allele and AA+AG genotypes of *ADAM33* T2 polymorphism was associated with increased the asthma risk in Asian populations (A vs. G: OR = 1.44, 95% CI = 1.13–1.85, *P* = 0.004; AA+AG vs. GG: OR = 1.45, 95% CI = 1.11–1.88, *P* = 0.006), not in Caucasian populations (A vs. G: OR = 0.93, 95% CI = 0.80–1.09, *P* = 0.38; AA+AG vs. GG: OR = 0.88, 95% CI = 0.67–1.15, *P* = 0.34). Subgroup analysis by ages demonstrated that A allele and AA+AG genotypes of T2 variant was associated with increased the childhood asthma risk (A vs. G: OR = 1.45, 95% CI = 1.11–1.89, P = 0.006; AA+AG vs. GG: OR = 1.43, 95% CI = 1.07–1.92, *P* = 0.02), not with adult patients (A vs. G: OR = 1.04, 95% CI = 0.83–1.29, *P* = 0.75; AA+AG vs. GG: OR = 1.03, 95% CI = 0.81–1.32, *P* = 0.79).

### Q1 (rs612709, G/A)

**Fig. 3:**
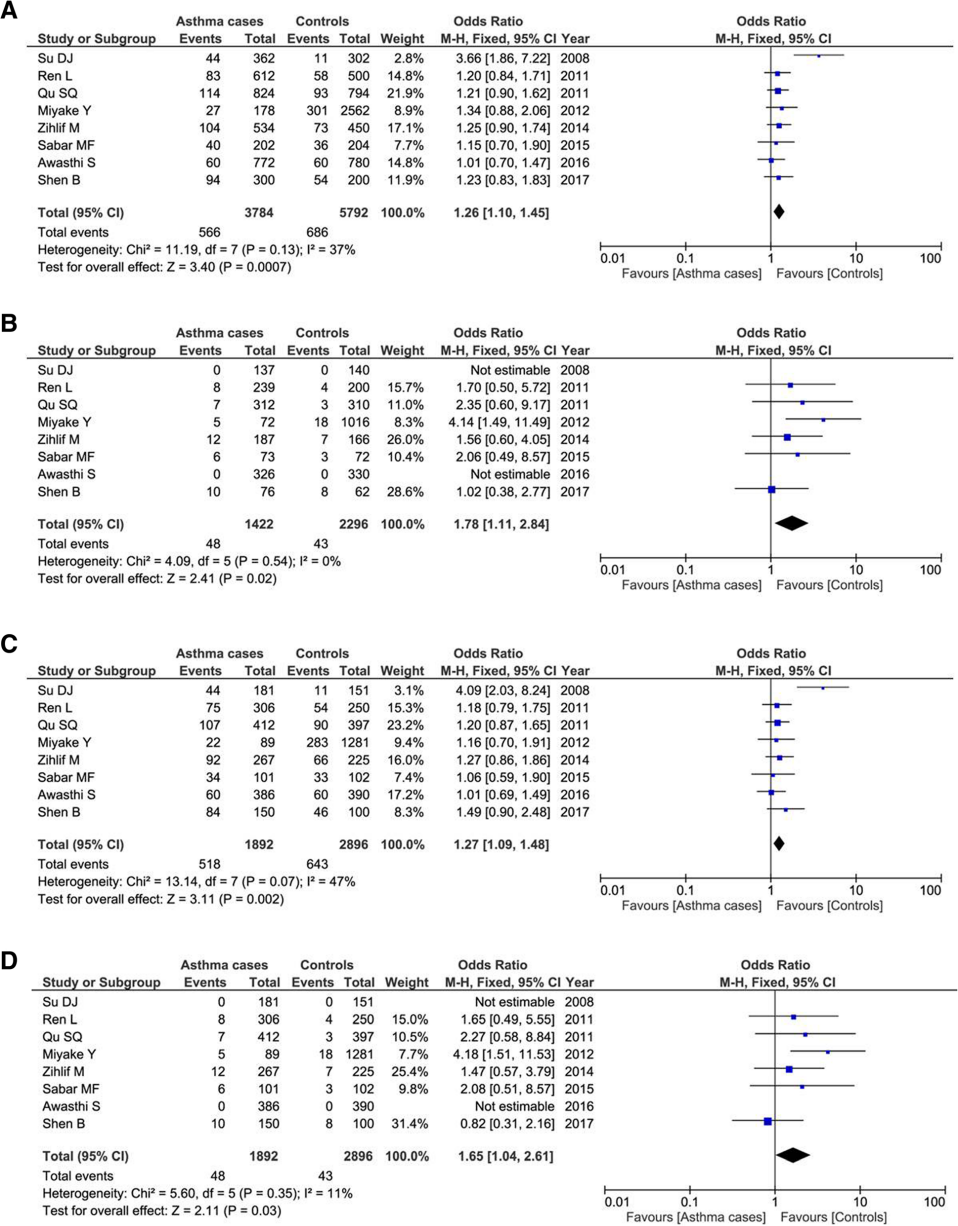
Forest plots of the association between *ADAM33* Q1 polymorphism and asthma risk in total population under the allelic model a, homozygous model b, dominant model c, and recessive model d

Eight relevant articles contained 1892 patients and 2896 controls. All the 8 articles were conducted in Asian population. Our result found Q1 polymorphism was related with increased the risk of asthma under four genetic comparison models (A vs. G: OR = 1.26, 95% CI = 1.10–1.45, *P* = 0.0007; AA vs. GG: OR = 1.78, 95% CI = 1.11–2.84, *P* = 0.02; AA+AG vs. GG: OR = 1.27, 95% CI = 1.09–1.48, *P* = 0.002; AA vs. AG + GG: OR = 1.65, 95% CI = 1.04–2.61, *P* = 0.03) as shown in Fig. 3. Subgroup analysis by ages showed that Q1 variant was not associated with childhood asthma.

### S1 (rs3918396, G/A)

Sixteen articles included 3756 asthma patients and 3630 controls. The statistical analysis showed that there was no significant association between S1 polymorphism and asthma risk under any genetic comparison models. This insignificance was also detected in Asians and Caucasians in subgroup analysis by ethnicities.

### S2 (rs528557, G/C)

Twenty-nine articles included 6492 patients and 7512 controls. Our analysis did not found a significant association between S2 polymorphism and asthma risk under any comparison models. Subgroup analysis by ethnicities and ages also did not find a significant relationship.

### S + 1 (r2853209, A/T)

Five articles included 1006 asthma patients and 2112 controls. No significant association was detected between S + 1 polymorphism and asthma risk.

### F + 1 (rs511898, C/T)

Twenty-two articles contained 5149 patients and 4795 controls. A significant association between F + 1 polymorphism and asthma risk was found under each comparison models (T vs. C: OR = 1.17, 95% CI = 1.06–1.30, *P* = 0.002; TT vs. CC: OR = 1.43, 95% CI = 1.14–1.80, P = 0.002; TC vs. CC: OR = 1.11, 95% CI = 1.01–1.21, *P* = 0.02; TT + TC vs. CC: OR = 1.15, 95% CI = 1.06–1.26, *P* = 0.001; TT vs. TC + CC: OR = 1.29, 95% CI = 1.05–1.59, P = 0.02). Subgroup analysis by ethnicities showed this genetic variant was associated with asthma in Asians (T vs. C: OR = 1.15, 95% CI = 1.03–1.28, *P* = 0.009; TT + TC vs. CC: OR = 1.13, 95% CI = 1.03–1.24, *P* = 0.01). Subgroup analysis by ages showed it was related with childhood asthma (T vs. C: OR = 1.26, 95% CI = 1.05–1.52, P = 0.01; TT + TC vs. CC: OR = 1.22, 95% CI = 1.07–1.39, *P* = 0.003).

### ST + 4 (rs44707, C/A)

Fifteen articles included 4096 patients and 3767 controls. Our result did not detect a significant association between ST + 4 variant and asthma risk in total population and subgroup analysis by ethnicities. However, subgroup analysis by ages showed that the A allele and AA+CA genotype was associated with childhood asthma (A vs. C: OR = 0.82, 95% CI = 0.69–0.97, *P* = 0.02; AA+CA vs. CC: OR = 0.81, 95% CI = 0.69–0.95, *P* = 0.010).

### ST + 5 (rs597980, C/T)

Six articles included 1535 patients and 1683 controls. No significant association was found between this genetic polymorphism and asthma risk.

### V4 (rs2787094, C/G)

Twenty-eight articles included 7108 patients and 7794 controls. Our result did not detect a significant association between V4 polymorphism and asthma risk. Subgroup analysis by ethnicities showed that it was associated with asthma risk in Caucasians (G vs. C: OR = 1.12, 95% CI = 1.00–1.26, *P* = 0.04; GG + CG vs. CC: OR = 1.17, 95% CI = 1.01–1.36, P = 0.04). Subgroup analysis by ages showed it was associated with asthma in adults (G vs. C: OR = 1.39, 95% CI = 1.04–1.84, *P* = 0.02; GG + CG vs. CC: OR = 1.52, 95% CI = 1.04–2.21, *P* = 0.03).

### Association between *ADAM33* genetic polymorphisms and asthma severity

According to the GINA guidelines, the confirmed asthma patients were divided into two group: the low-severity group included patients with intermittent or mild persistent asthma attacks; the high-severity group consisted of patients with moderate or severe persistent asthma attacks. Several included case-control studies considered the *ADAM33* polymorphisms in asthma severity.

**Fig. 4:**
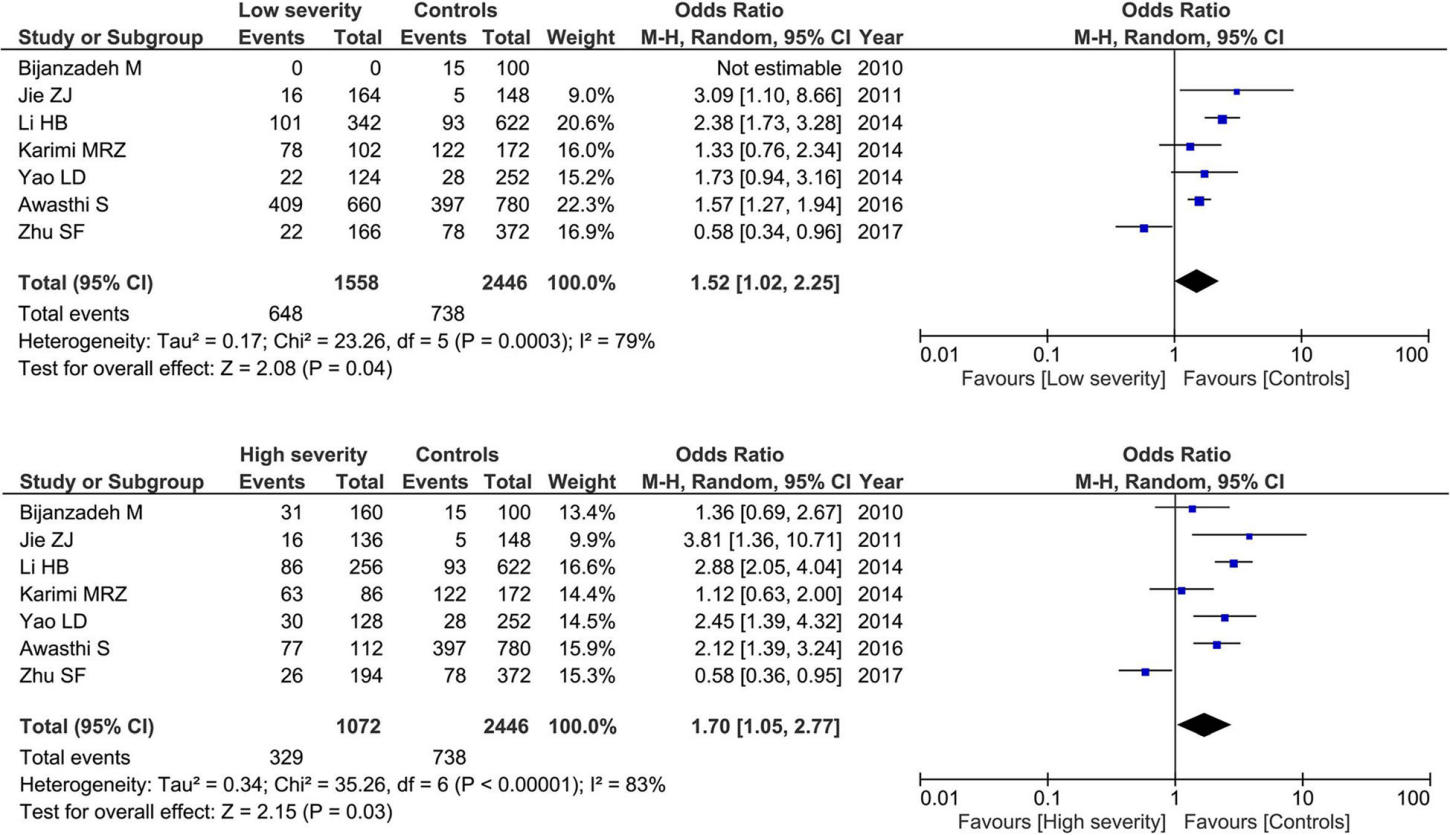
Subgroup analysis by asthma severity for the association between *ADAM33* T1 polymorphism and asthma risk under the allele model

For T1 variant, 7 articles contained 1315 patients and 1223 control. Our result found that only the G allele of *ADAM33* T1 polymorphism was associated with low-severity (G vs. A: OR = 1.52, 95% CI = 1.02–2.25, *P* = 0.04) and high-severity asthma (G vs. A: OR = 1.70, 95% CI = 1.05–2.77, *P* = 0.03) when compared with the controls as shown in Fig. 4. No significant difference was noted between the high- and low- severity groups in the allele frequencies in this genetic polymorphism. The GG + GA genotypes was not associated with asthma severity in low-severity group (GG + GA vs. AA: OR = 1.60, 95% CI = 0.87–2.92, *P* = 0.13) or high-severity group (GG + GA vs. AA: OR = 1.77, 95% CI = 0.94–3.35, *P* = 0.08).

For T2 variant, 8 articles contained 1539 patients and 1466 controls. No significant association was found between the allele and genotype of T2 variant and asthma severity (A vs. G:OR = 1.53, 95% CI = 0.86–2.70, *P* = 0.15; AA+AG vs. GG: OR = 1.42, 95% CI = 0.74–2.73, *P* = 0.29 for the low-severity group and A vs. G:OR = 2.05, 95% CI = 0.80–5.26, *P* = 0.14; AA+AG vs. GG: OR = 2.37, 95% CI = 0.80–6.99, *P* = 0.12 for the high-severity group).

For S2 variant, 8 articles contained 1398 patients and 1434 controls. Our result showed that this genetic variant was not associated with asthma severity (C vs. G: OR = 1.44, 95% CI = 0.79–2.62, *P* = 0.24; CC + GC vs. GG: OR = 1.51, 95% CI = 0.57–3.95, *P* = 0.41 for the low-severity group; C vs. G: OR = 1.47, 95% CI = 0.86–2.51, *P* = 0.16; CC + GC vs. GG: OR = 1.27, 95% CI = 0.60–2069, *P* = 0.53 for the high-severity group).

For V4 variant, 8 articles contained 1269 patients and 1347 controls. No significant association was detected (G vs. C: OR = 0.91, 95% CI = 0.60–1.37, *P* = 0.65; GG + CG vs. CC OR = 1.00, 95% CI = 0.66–1.51, *P* = 0.99 for the low-severity group; G vs. C: OR = 1.13, 95% CI = 0.60–2.15, *P* = 0.70; GG + CG vs. CC OR = 1.11, 95% CI = 0.54–2.27, *P* = 0.78 for the high-severity group).

### Association between *ADAM33* polymorphisms and asthma risk in Chinese population

Thirty-four case-control studies were conducted in Chinese population, including 6136 asthma patients and 5016 controls. Table 3 listed the summary analysis of *ADAM33* genetic polymorphisms and asthma risk among Chinese population. Our result showed that the alleles and genotypes of *ADAM33* T2 (A vs. G: OR = 1.70, 95% CI = 1.33–2.17, *P* < 0.0001; AA+AG vs. GG: OR = 1.71, 95% CI = 1.33–2.21, P < 0.0001), Q1 (A vs. G: OR = 1.45, 95% CI = 1.01–2.07, *P* = 0.04; AA+AG vs. GG: OR = 1.58, 95% CI = 1.02–2.43, P = 0.04), and F + 1 (T vs. C: OR = 1.13: OR = 1.03–1.24, *P* = 0.01; TT + TC vs. CC: OR = 1.15: OR = 1.02–1.30, *P* = 0.02) variants were significantly associated with increased the asthma risk in Chinese asthma patients. This statistically significance was not detected in other genetic polymorphisms.

**Table 3:** Meta-analysis of the association between the alleles and genotypes of ADAM33 genetic polymorphisms and asthma risk in Chinese population

SNPs	Comparison	N	Test of association		Test of heterogeneity		
			OR (95%CI)	P	Ph	I^2^(%)	Model
T + 1 (rs2280089, G/A)	A vs. G	3	1.65 (0.73, 3.74)	0.23	< 0.0001	91	R
	AA+AG vs. GG		1.79 (0.70, 4.56)	0.22	< 0.0001	91	R
T1 (rs2280091, A/G)	G vs. A	15	1.36 (0.91, 2.02)	0.13	< 0.00001	91	R
	GG + AG vs. AA		1.45 (0.92, 2.27)	0.11	< 0.00001	90	R
T2 (2,280,090, G/A)	A vs. G	18	1.70 (1.33, 2.17)	< 0.0001	< 0.00001	79	R
	AA+AG vs. GG		1.71 (1.33, 2.21)	< 0.0001	< 0.00001	77	R
Q1 (rs612709, G/A)	A vs. G	4	1.45 (1.01, 2.07)	0.04	0.02	68	R
	AA+AG vs. GG		1.58 (1.02, 2.43)	0.04	0.01	72	R
S1 (rs3918396, G/A)	A vs. G	5	1.00 (0.80, 1.25)	0.99	0.33	14	F
	AA+AG vs. GG		1.12 (0.70, 1.78)	0.64	0.44	0	F
S2 (rs528557, G/C)	C vs. G	17	0.89 (0.76, 1.04)	0.13	< 0.00001	70	R
	CC + GC vs. GG		0.86 (0.66, 1.14)	0.37	< 0.0001	67	R
F + 1 (rs511898, C/T)	T vs. C	13	1.14 (1.04, 1.25)	0.004	0.50	0	F
	TT + TC vs. CC		1.15 (1.02, 1.30)	0.02	0.32	12	F
ST + 4 (rs44707, C/A)	A vs.C	5	0.96 (0.75, 1.23)	0.75	0.01	69	R
	AA+CA vs. CC		0.92 (0.68, 1.24)	0.58	0.06	56	R
ST + 5 (rs597980, C/T)	T vs. C	3	0.86 (0.73, 1.02)	0.08	0.79	0	F
	TT + CT vs. CC		0.83 (0.66, 1.04)	0.11	0.76	0	F
V4 (rs2787094, C/G)	G vs. C	11	1.12 (0.73, 1.71)	0.60	< 0.00001	95	R
	GG + CG vs. CC		1.10 (0.63, 1.91)	0.74	< 0.00001	92	R

*SNPs* single nucleotide polymorphisms, *N* number of included studies, *OR* odds ratio, *95% CI* 95% confidence interval, *Ph* p-value of heterogeneity, *R* random-effect model, *F* fixed-effect model

### Sensitivity analysis and publication bias

**Fig. 5:**
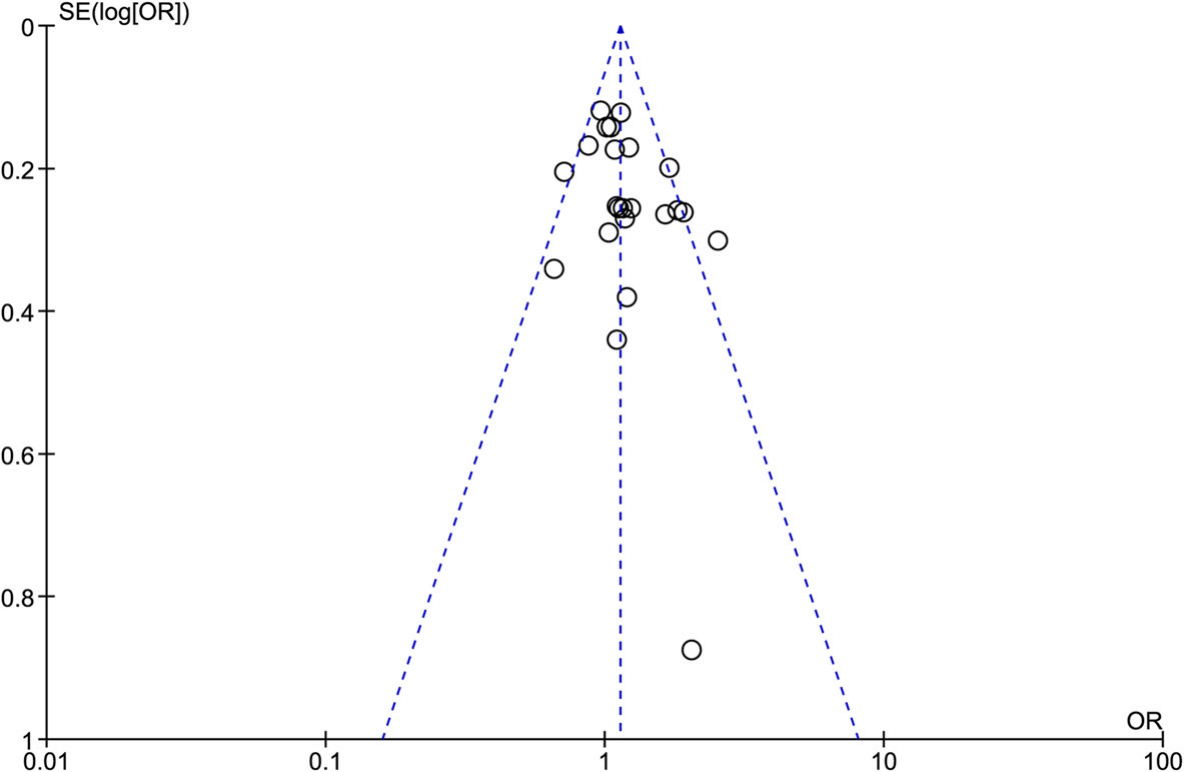
Funnel plot of *ADAM33* F + 1 polymorphism and asthma risk under the domain model

We conducted a sensitivity analysis to assess if our results were substantially affected by the presence of any individual study. Each included case-control study was deleted at each time to recalculate the significance of the results. Our result suggested that the pooled ORs were not significantly changed. The funnel plot was applied to evaluate the publication bias, and the shape of the funnel plot was symmetrical, further indicating that no publication bias existed in this meta-analysis as shown in Fig. 5.

## Discussion

In this meta-analysis, we totally screened out 63 case-control articles. Our results found that T2, Q1 and F + 1 polymorphisms of *ADAM33* gene were associated with asthma risk in total population. Subgroup analysis by ethnicities showed that the alleles and genotypes of T2, Q1 and F + 1 polymorphisms were associated with asthma susceptibility among Asian populations, while V4 polymorphisms was related with asthma risk among Caucasian populations. Subgroup analysis by ages showed that T2, F + 1 and ST + 4 polymorphisms were associated with childhood asthma, while Q1 and V4 were associated with asthma risk in adults. Subgroup analysis by asthma severity showed that the G allele of *ADAM33* T1 polymorphism was associated with the severity of asthma when compared with the controls. In addition, T2, Q1 and F + 1 polymorphisms of *ADAM33* were significantly associated with increased the asthma risk in Chinese asthma patients. Our results were inconsistence with previous meta-analyses conducted by Liu et al. which found that S2 polymorphism may contribute to asthma susceptibility [91]; Deng et al. which suggested that T1 polymorphism might be a potential susceptible predictor of asthma for Asian children [92]; Zheng et al. which showed that *ADAM33* V4 polymorphism contributes to the risk of asthma and may be utilized as a biomarker for the early diagnosis of asthma [93]; Sun et al. which demonstrated that *ADAM33* polymorphisms T1, T2, V4, S2 and ST + 4 were significantly associated with a high risk of childhood asthma [94].

Asthma, caused by obstruction of the conducting airways of the lung, is a chronic inflammatory disease in which cells of the innate and adaptive immune systems act together with epithelial cells, leading to mucus overproduction, bronchial hyperreactivity, airway wall remodeling and airway narrowing [95, 96]. It is a complex disease influenced by the interaction of gene-gene, gene-protein and protein-protein, and the environmental issue, which plays a significant role in causing asthma pathogenesis and makes it difficult to define precisely in population studies. Because of differences in the influence of genes and environment, there is a wide range of disease heterogeneity and severity in asthma [97]. Identification and measurement of significant biomarkers are particularly crucial in clinical research of asthma to characterize the population and to associate the disease with therapeutic effects [98].

ADAM33, a membrane-anchored protease expressed in multiple airway cell types, is known to be an important gene involved in asthma pathogenesis. It is upregulated during acute or chronic lung inflammation, and recent functional and genetic analyses have linked it to disease development [99]. Studies have found that ADAM33 can be detected as sADAM33 in asthmatic airways in which high levels of sADAM33 are correlated with reduced lung function [100]. The expression of serum ADAM33 was up-regulated in asthmatic patients along with the severity of asthma [101]. In addition, ADAM33 was positively correlated with serum IL-4 and IL-13, implying that the expression of ADAM33 may be regulated by Th2 type cytokines [101]. The identification of *ADAM33* as a major risk factor involved in the pathogenesis of BHR and airway wall remodeling provides insight into the pathogenesis of asthma and represents a novel therapeutic target [102]. Taken together, it can be concluded that ADAM33 mediates the airway inflammatory and remodeling effects and is a potential therapeutic target for asthma.

*ADAM33* is one of the positionally cloned asthma associated genes, which has been identified as susceptibility gene for asthma in different populations. Genetic polymorphism is the occurrence of two or more clearly different morphs or forms, also referred to as alternative phenotypes, in the population of a species, thus involving in disease progression. Van Eerdewegh et al. performed a genome-wide scan on 460 Caucasian families and firstly identified the *ADAM33*, a putative asthma susceptibility gene identified by positional cloning in an outbred population, provided insights into the pathogenesis and natural history of asthma. Subsequently, a large number of related studies have been carried out. However, the results were inconclusive in different populations, even in the same population. The study conducted by Werner et al. evaluated the association between *ADAM33* polymorphisms and asthma and related traits in two German populations, and replicated the results of Van Eerdewegh et al., but most of the associated SNPs were at non-identical positions in the German, UK and US samples [103]. Jongepier et al. found that *ADAM33* S2 variant was shown to be associated with the development of asthma and disease progression, possibly related to enhanced airway remodeling in Dutch Caucasian individuals [104]. Noguchi et al. surveyed 155 families and confirmed the involvement of *ADAM33* in the development of childhood asthma among the Japanese [105]. Lind et al. studied 659 mild or moderate-severe subjects, and concluded that the *ADAM33* gene was not an important risk factor for asthma or asthma-associated phenotypes in Mexicans or in Puerto Ricans [106]. Kedda et al. investigated the role of 10 SNPs of *ADAM33* gene in asthma risk and asthma severity in Australian Caucasian population, and did not detect significant association between any one of the SNPs and asthma or asthma severity, but there was a significant global haplotype association with asthma and disease severity [107]. Murk et al. found that SNPs in frequently replicated asthma risk genes *ADAM33* revealed no association [108]. Blakey et al. conducted the analysis in a large, nationally representative, white ethnicity population (*n* = 7703) and found that S2, not F + 1 polymorphism of *ADAM33* influenced asthma risk in the UK population [109]. Although several meta-analyses had been conducted to identify the role of *ADAM33* variants in asthma risk, the results still remain inconclusive [110]. Therefore, we conducted the present meta-analysis.

China is the world’s largest developing country and has the largest population. The prevalence of asthma significantly varies among different regions of China. Although the incidence rate of asthma is lower in Chinese children and adults than that in developed countries, the prevalence has been on the rise during the past three decades. Besides, 40% of asthma patients in China are uncontrolled, resulting in the increase of hospitalization, emergency room visit and absence of work or school [111]. Even though asthma control has been improved in some cities, the level of asthma control in China is still far from ideal at present [112]. Environmental exposure and genetic predisposition were identified as risk factors for asthma in China [113]. In our study, we found that T2, Q1 and F + 1 polymorphisms of *ADAM33* were significantly associated with increased the asthma risk in Chinese asthma patients, indicating that *ADAM33* might be a promising biomarker and therapeutic option for asthma.

In addition, several studies focused on *ADAM33* as a major hub and identified some proteins whose interaction with ADAM33 had been associated with asthma. Kong et al. found that the combination of *ADAM33*, *Smad7,* and *LIGHT* would be a reliable and useful childhood asthma model for prediction and diagnosis [114]. Vishweswaraiah et al. recognized amyloid β (A4) precursor protein, ataxin-7, α4-integrin, and α5-integrin, which were involved in airway hyperresponsiveness, and through the interaction with ADAM33, they might have potential relevance in asthma [115]. Sangeetha et al. identified few proteins, such as APP, ATXN7, ITGA4, and ITGA5, whose interaction with the ADAM33 might have been associated with asthma [116]. Besides, *ADAM33* might also interact with other environmental factors. Bukvic et al. demonstrated several novel significant interactions between polymorphisms in 20p13-p12 and early-life environmental tobacco smoke exposure with asthma presence and, amongst asthmatics, a significant association with the severity of their disease [117].

Several limitations were presented in our meta-analysis. Firstly, half of the included articles were conducted in Chinese population, while other populations should be discussed. Secondly, the between-study heterogeneity was detected in most genetic comparison models, which might influence our results. It probably because these included articles were conducted in different geographical regions, and asthma patients might receive different treatment, which might influences the asthma phenotype, leading to heterogeneity. Thirdly, the haplotypes among these SNPs should be considered because identifying these statistical associations and few alleles of a specific haplotype sequence can facilitate identifying all other such polymorphic sites that are nearby on the chromosome. Lastly, interaction of gene-gene and gene-environmental factors should be considered in future studies.

## Conclusions

In conclusions, our results demonstrated that T2, Q1 and F + 1 polymorphisms of *ADAM33* gene might be risk factors for asthma susceptibility. Moreover, T2, Q1 and F + 1 polymorphisms were associated with asthma susceptibility among Asian populations, whereas V4 polymorphism was associated with asthma among Caucasian populations; T2, F + 1 and ST + 4 polymorphisms were associated with childhood asthma, whereas Q1 and V4 were associated with asthma risk in adults; T1 G allele polymorphism was associated with low-severity and high-severity asthma risk. Besides, *ADAM33* T2, Q1 and F + 1 polymorphisms were significantly associated with increased the asthma risk in Chinese asthma patients. Future case-control studies with large populations are still needed.

## Abbreviations

*ADAM33*: A disintegrin and metalloprotease 33
BHR: Bronchial hyper-responsiveness
CI: Confidence interval
MeSH: Medical Subject Heading
OR: Odds ratio
SNP: Single nucleotide polymorphism
UTR: Untranslated region
